# Supplementary material for: Tumor γδ T-cell abundance is associated with favorable cancer treatment outcomes
Source: bioRxiv. 2026 Sep 1:2026.08.27.747587. Preprint. [Version 1] doi: 10.64898/2026.08.27.747587 (PMC13684651; doi:10.64898/2026.08.27.747587)
Supplement: Supplementary file 1 [file NIHPP2026.08.27.747587v1-supplement-1.pdf]

bioRxiv preprint doi: https://doi.org/10.64898/2026.08.27.747587; this version posted September 1, 2026. The copyright holder for this preprint (which was not certified by peer review) is the author/funder, who has granted bioRxiv a license to display the preprint in perpetuity. It is made available under a CC-BY 4.0 International license.

Supplementary figures

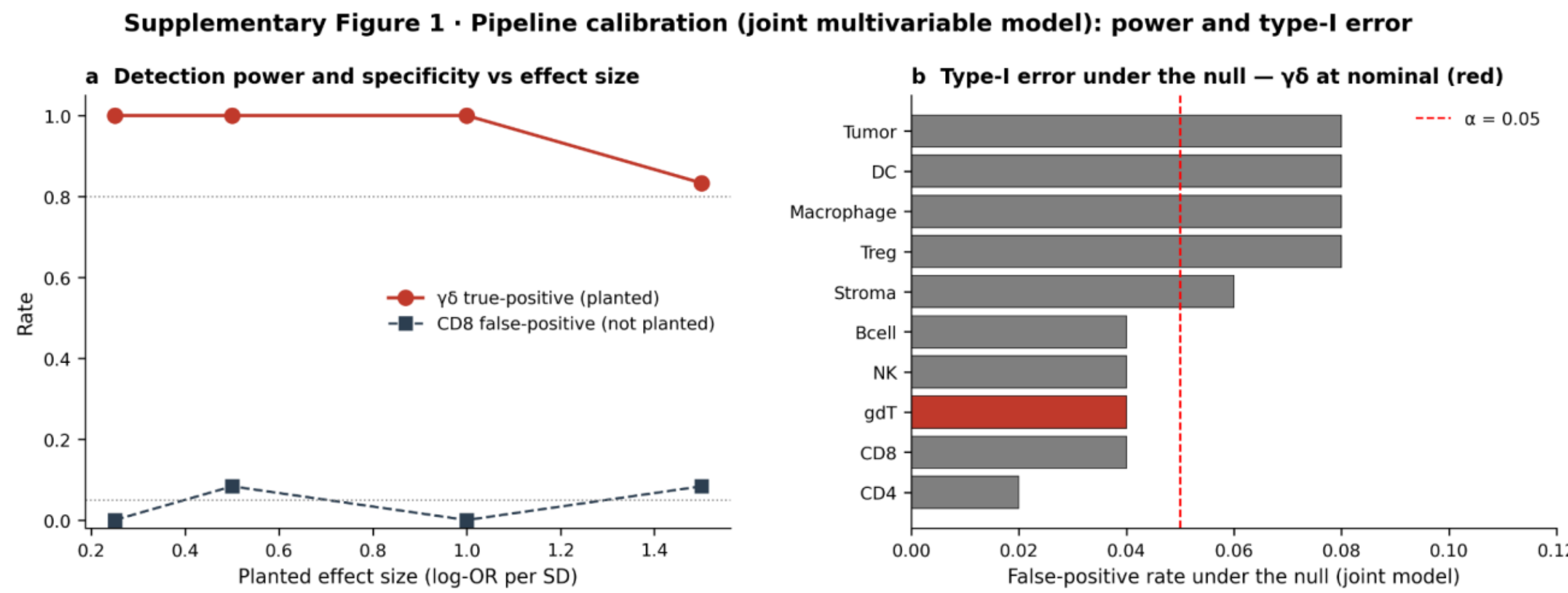

Fig. S1. Pipeline calibration (joint multivariable model).
(a) Detection power and specificity versus planted effect size: simulated γδ effects are recovered at approximately 100% power across realistic effect sizes, while the false-positive rate for a non-planted CD8 signature stays near α. (b) Type-I error under the global null: the γδ false-positive rate is at the nominal level (0.04 at α = 0.05), with no γδ-specific inflation. Pseudo-bulk simulation design in Methods §2.4.

bioRxiv preprint doi: https://doi.org/10.64898/2026.08.27.747587; this version posted September 1, 2026. The copyright holder for this preprint (which was not certified by peer review) is the author/funder, who has granted bioRxiv a license to display the preprint in perpetuity. It is made available under a CC-BY 4.0 International license.

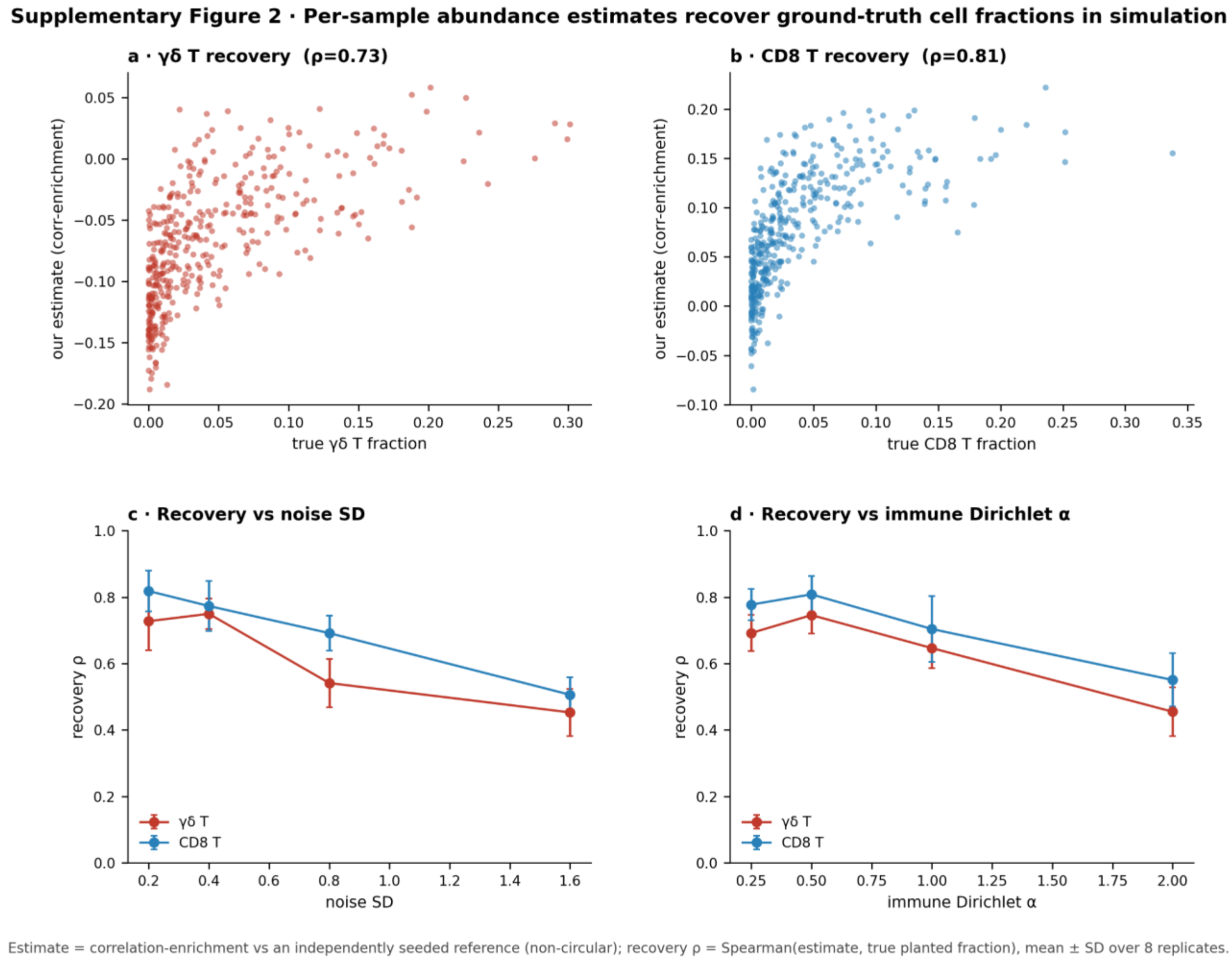

Fig. S2. Per-sample abundance scores recover ground-truth cell fractions in simulation.

Pseudo-bulk samples were built from known cell-type mixtures and scored by correlation-enrichment against an independently seeded reference (non-circular). (a, b) Recovery of the planted γδ and CD8 T-cell fractions (Spearman $\rho = 0.73$ and 0.81). (c, d) Recovery as a function of added noise (c) and mixture concentration (Dirichlet α; d); values are mean ± SD over 8 replicates. Methods §2.4.

bioRxiv preprint doi: https://doi.org/10.64898/2026.08.27.747587; this version posted September 1, 2026. The copyright holder for this preprint (which was not certified by peer review) is the author/funder, who has granted bioRxiv a license to display the preprint in perpetuity. It is made available under a CC-BY 4.0 International license.

bioRxiv preprint doi: https://doi.org/10.64898/2026.08.27.747587; this version posted September 1, 2026. The copyright holder for this preprint (which was not certified by peer review) is the author/funder, who has granted bioRxiv a license to display the preprint in perpetuity. It is made available under a CC-BY 4.0 International license.

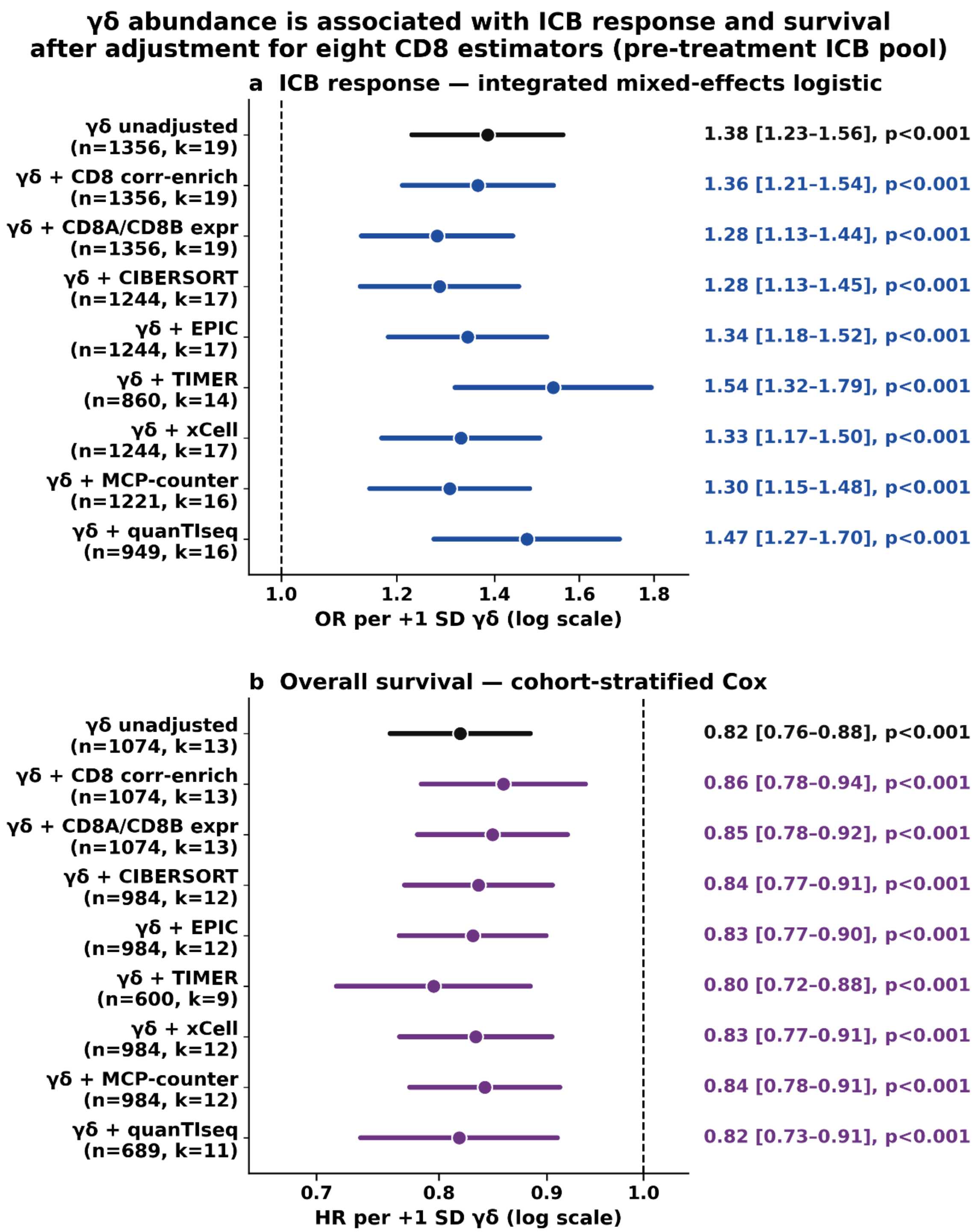

Dot = point estimate, bar = 95% CI, dashed line = no effect. n = samples, k = cohorts contributing to that row. Response model = binomial mixed model with a random intercept per cohort; survival model = Cox stratified by cohort. γδ and each CD8 estimator are z-scored within cohort. CIBERSORT/EPIC/xCell/MCP-counter, TIMER and quanTIseq are not available for every cohort, so n and k shrink accordingly.

Fig. S3. γδ abundance is associated with ICB response and survival after adjustment for eight CD8 estimators.

bioRxiv preprint doi: https://doi.org/10.64898/2026.08.27.747587; this version posted September 1, 2026. The copyright holder for this preprint (which was not certified by peer review) is the author/funder, who has granted bioRxiv a license to display the preprint in perpetuity. It is made available under a CC-BY 4.0 International license.

Pooled analysis of pretreatment ICB cohorts (19 cohorts, n = 1,356 for response; 13 cohorts, n = 1,074 for OS). (a) ICB response (binomial mixed-effects logistic): γδ odds ratio per +1 SD, shown unadjusted (top row) and after adding each of the eight CD8 estimators as a covariate; all remain significant (OR 1.28–1.54, $p < 0.001$). (b) Overall survival (cohort-stratified Cox): γδ hazard ratio per +1 SD, unadjusted and CD8-adjusted (HR 0.80–0.86, $p < 0.001$). γδ and each CD8 estimator are z-scored within cohort; CIBERSORT/EPIC/xCell/MCP-counter, TIMER, and quanTIseq are not available for every cohort, so n and k vary by row. Methods §2.4.

bioRxiv preprint doi: https://doi.org/10.64898/2026.08.27.747587; this version posted September 1, 2026. The copyright holder for this preprint (which was not certified by peer review) is the author/funder, who has granted bioRxiv a license to display the preprint in perpetuity. It is made available under a CC-BY 4.0 International license.

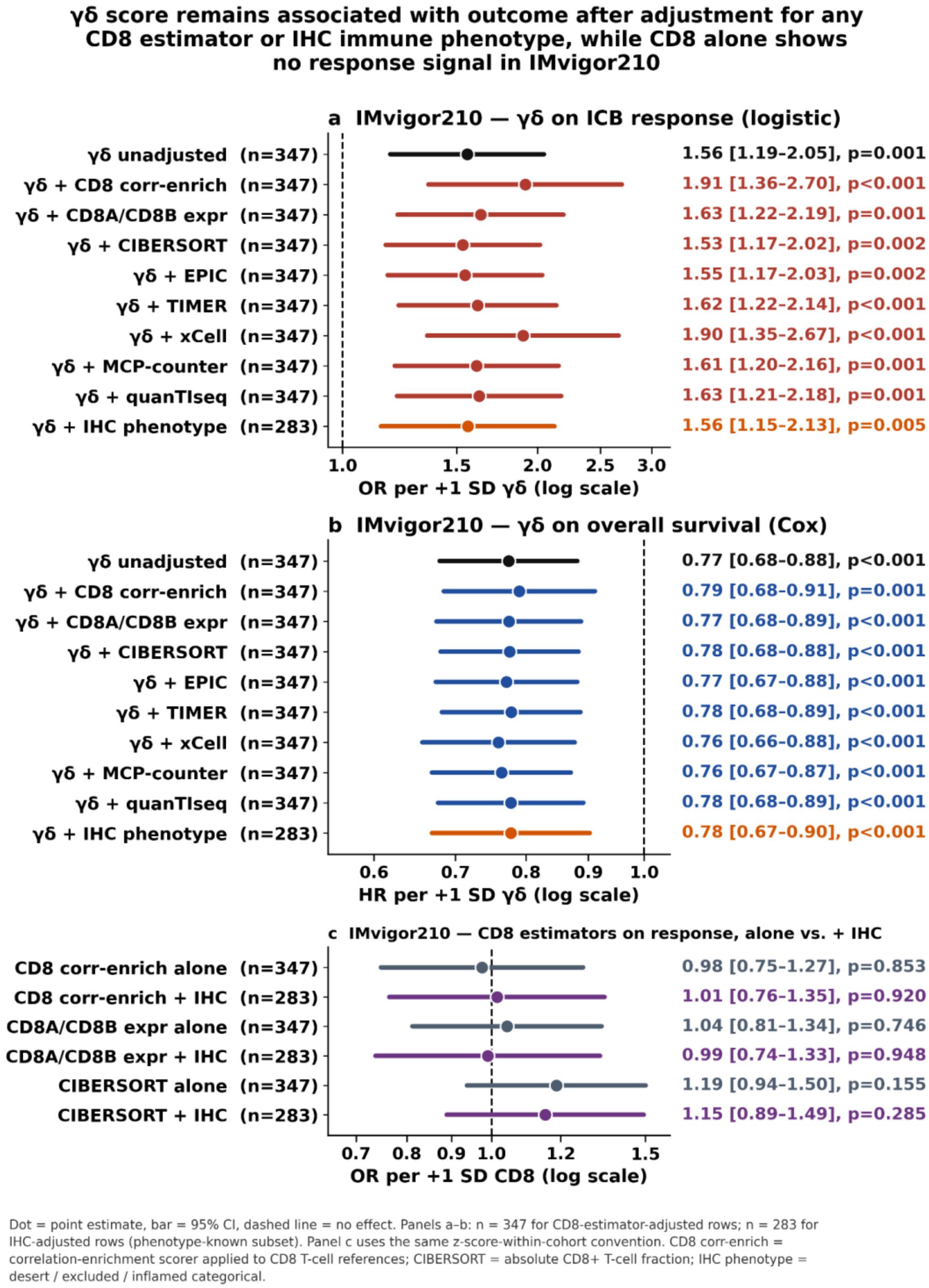

Fig. S4. In IMvigor210, the γδ–outcome association remains after adjustment for each CD8 estimator and the orthogonal IHC immune phenotype, while CD8 alone is uninformative.

Atezolizumab-treated urothelial cohort (n = 347; IHC immune phenotype available for n = 283). (a) ICB response (logistic): γδ odds ratio per +1 SD, unadjusted (top row) and after adding each of eight CD8 estimators or the IHC phenotype as a covariate; all remain above 1 and significant (OR

bioRxiv preprint doi: https://doi.org/10.64898/2026.08.27.747587; this version posted September 1, 2026. The copyright holder for this preprint (which was not certified by peer review) is the author/funder, who has granted bioRxiv a license to display the preprint in perpetuity. It is made available under a CC-BY 4.0 International license.

1.53–1.91; every $p < 0.005$). (b) Overall survival (Cox): γδ hazard ratio per +1 SD, unadjusted and after the same adjustments (HR 0.76–0.79; every $p < 0.005$). (c) Specificity control: each CD8 estimator was fitted as the predictor in place of γδ, alone and after IHC-phenotype adjustment; CD8 carries no response signal (OR 0.98–1.19; all $p > 0.15$), so the γδ effect is not a relabeled CD8 effect. γδ and CD8 are z-scored within cohort. Methods §2.4.

bioRxiv preprint doi: https://doi.org/10.64898/2026.08.27.747587; this version posted September 1, 2026. The copyright holder for this preprint (which was not certified by peer review) is the author/funder, who has granted bioRxiv a license to display the preprint in perpetuity. It is made available under a CC-BY 4.0 International license.

Per-cohort γδ score by ICB response
(R = responders, NR = non-responders; pre-treatment)

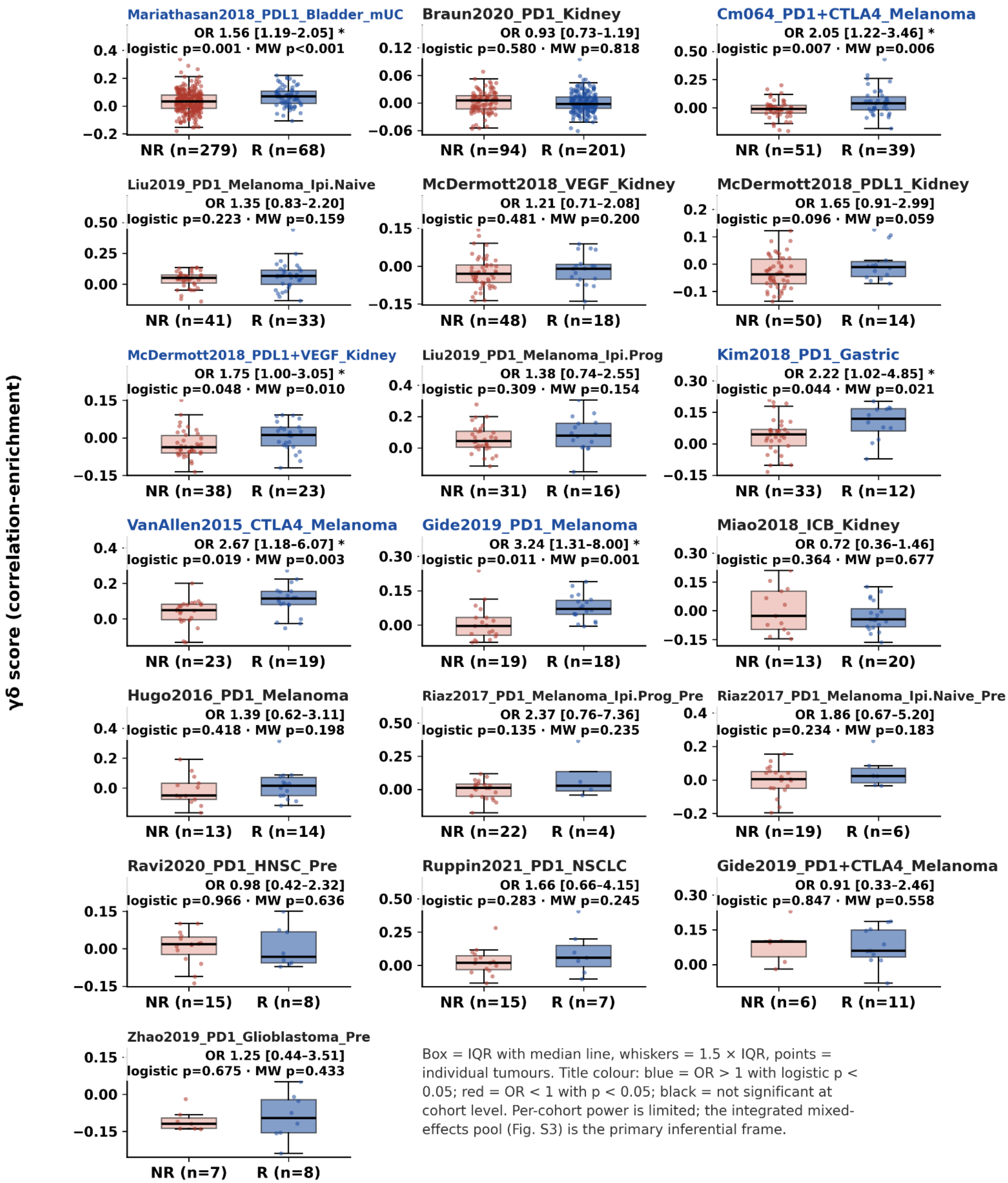

Box = IQR with median line, whiskers = 1.5 × IQR, points = individual tumours. Title colour: blue = OR > 1 with logistic p < 0.05; red = OR < 1 with p < 0.05; black = not significant at cohort level. Per-cohort power is limited; the integrated mixed-effects pool (Fig. S3) is the primary inferential frame.

bioRxiv preprint doi: https://doi.org/10.64898/2026.08.27.747587; this version posted September 1, 2026. The copyright holder for this preprint (which was not certified by peer review) is the author/funder, who has granted bioRxiv a license to display the preprint in perpetuity. It is made available under a CC-BY 4.0 International license.

Fig. S5. Per-cohort γδ score by ICB response.

Per-cohort distribution of the per-sample γδ correlation-enrichment score in non-responders (NR) versus responders (R), one panel per ICB cohort with response data (19 cohorts, pretreatment). Each title gives that cohort's logistic odds ratio (per +1 SD γδ) and Mann–Whitney p; blue titles mark a significant favorable association (OR > 1, $p < 0.05$), black titles denote nonsignificant results. γδ was higher in responders (OR > 1) in 15 of 19 cohorts, reached per-cohort significance in 6 (Mariathasan, CheckMate-064, Gide PD-1, Van Allen, Kim, McDermott PD-L1+VEGF), and no cohort showed a significant adverse association; individual cohorts are underpowered, so the integrated mixed-effects pool (Fig. S3) is the primary inferential framework. Methods §2.4.
